# Determinants of Consumers’ Trust in Biotech Brands and Purchase Intentions towards the Cord Blood Products

**DOI:** 10.3390/ijerph182111574

**Published:** 2021-11-04

**Authors:** Shih-Wei Chen, Ku-Yuan Lee, Chi-Ming Hsieh

**Affiliations:** 1 Department of Otolaryngology, Tungs’ Taichung MetroHarbor Hospital, Taichung 43503, Taiwan; eddielodz@gmail.com; 2 Ph.D. Program in Translational Medicine, Department of Life Sciences, National Chung Hsing University, Taichung 40227, Taiwan; 3 College of Intelligence, National Taichung University of Science and Technology, No. 129, Sec. 3, Sanmin Rd., North Dist., Taichung 40401, Taiwan; 4 International Bachelor Program of Agribusiness, National Chung Hsing University, 145 Xingda Rd., South Dist., Taichung 40227, Taiwan

**Keywords:** trust, biotech industry, brand, purchase intention

## Abstract

The development of the biotech industry is in full swing, and consumers have begun to value biotech brands. Since biotech products often focus on the future or special benefits, consumers inevitably bear certain risks when purchasing biotech products, and their trust in the biotech brand will have an important impact on their purchase intention. Previous studies have lacked a targeted understanding of consumer trust in biotech brands and a discussion of cultural viewpoints. This study introduced the concept of personal connections in Chinese relationalism and trust strategies in Chinese society to address this gap. In-depth interviews and focus group discussions were conducted in collaboration with Company X, a listed Taiwanese cord blood company, to extract the key factors influencing consumer trust and purchase intention of biotech brands. After constructing the structure model, the study was validated using a structural equation model through investigation and survey. The findings indicated that consumer trust in biotech brands was constructed by a combination of kinship trust transfer and emergent trust transfer within the consumer relationship network, as well as institutional trust and professional trust outside the relationship network and that a significant positive correlation existed between consumer trust in biotech brands and purchase intention. The acquaintances within the consumer relationship network include not only relatives and friends but also health care workers and netizens that consumers come into contact with. In addition, kinship trust transfer and emergent trust transfer within the consumer relationship network have a greater impact on trust in biotech brands than the institutional trust and professional trust outside the relationship network. The findings of this study deepen the understanding of consumer trust in biotech brands across cultures, and suggest that future marketing communication should be expanded to include key players within the consumer relationship network.

## 1. Introduction

The biotech industry is a major industry that Taiwan has been developing in recent years. Since the inception of the “Taiwan Biotech Industry Take-Off Diamond Action Plan” in 2009, the investment and development of Taiwan’s biotechnology industry have been in full swing with the policy announcement of the Executive Yuan. In 2013, the Executive Yuan again promoted the “Taiwan Biotech Industry Take-Off Action Plan,” which pushed Taiwan’s biotech industry from “efficiency-oriented” to “open innovation” and shifted the focus of development from medical devices to health care services. In 2017, the “Biomedical Industry Innovation Promotion Program” further highlighted “precision health,” and the concept of health care advanced from the treatment of diseases to the goals of prevention and prediction [1]. Since its development, the biotech industry has become a new favorite for investment, and the number of newly listed companies has also increased, which has a growing impact on public health care. Such a process shows that the development of Taiwan’s biotech industry has shifted its focus from B2B to B2C, and the priority of health care products has shifted to consumers’ future prevention and care.

With the rapid growth of the B2C market in the biotech industry, a company’s ability to increase consumer purchase intention is a key factor in its growth. A company that fails to successfully enhance its brand trust and thereby increase consumer intention to purchase its health care products will lose out to consumers. Today’s health care products, such as cord blood, deciduous tooth stem cells, and genetic testing, mostly highlight future health protection, potential disease risks, and unknown genetic diseases. This makes it impossible for consumers to immediately judge the quality of the product when purchasing a health care product and confirm whether the biotech company will grow as expected. As a result, consumers are often in a risky situation when purchasing health care products, especially when it comes to time risk. A future defect in the product, an accident before the actual time of use, or the future bankruptcy of the company could wipe out the consumer’s investment. Such a risky situation can easily discourage consumers from purchasing health care products. In a risky situation, price is often not the most important consideration for consumers, but trust is [2]. Trust is the pivotal factor for someone to be willing to interact with others because they believe that others are reliable when they need to make decisions under uncertainty in a risky situation [3].

Whether a biotech brand is trusted by consumers will determine their willingness to purchase its health care products. Therefore, in practice, many biotech companies have regarded the establishment of their biotech brands as the emphasis of their growth and attempted to build consumer trust in the biotech brands [4]. BIONET, a cord blood company in Taiwan, mainly sells the cord blood products which are rich in stem cells used to treat blood diseases, innate metabolic diseases, and immune deficiency. BIONET mainly sell the products to the families with newborns. BIONET strengthens consumers’ recognition of its system by listing on the stock exchange and further enhances its brand image of professionalism and scale in consumers’ minds through professional division of labor before listing and professional certification (e.g., American Association of Blood Banks (AABB) certification). In the case of HealthBaby, the bank emphasized celebrity endorsement to enhance consumers’ sense of professionalism in the brand; as regards StemCell, the bank emphasized the size of its capital and enhanced consumer trust through institutional protection; for StemCyte, the bank emphasized its unique American business and improved its professional image through US certification, thus building up consumer trust.

The branding strategies in the above practices all stress enhancing consumer trust in their brands, proving that the industry has realized that trust in biotech brands and consumer purchase intention are significant cornerstones for the growth of biotech companies. However, in past studies of the biotech industry, only a few have discussed the influencing factors of consumer purchase intention [5,6]. There is a lack of not only an in-depth understanding of consumer purchase intention in the biotech industry but also adequate discussion on the key variable of trust in biotech brands. Consequently, the biotech industry is often limited in practical application by past business experience without sufficient empirical support and is easily caught in the limitations of its own experience [7].

Further, although many studies have shown that trust has significant positive effects on consumer purchase intention [7] and empirical evidence is also available in the risky context of Taiwan [8,9,10], Fukuyama et al.’s study on trust theory indicated that cultural background is the basis for individuals’ perceptions and judgments of the social environment in which they live and that different cultures produce different key factors to influence individuals’ trust formation [11]. In the above-mentioned empirical studies in Taiwan, however, the factors in trust formation are all directly adopted from foreign trust studies, which leads to a lack of cultural differences and localization in the understanding of trust in related studies. Therefore, this study introduces the concept of personal connections in Chinese relationalism and trust strategies in Chinese society to investigate the key factors that influence consumer trust and purchase intention of biotech brands in Taiwan from various cultural perspectives. Lo and Yeh [12] revealed that the Chinese trust formation mechanism in economic transactions differs from that of Western societies, and it is based on relationalism that produces personal connections of concentric or differential patterns. The degree of trust varies with the closeness of personal connections, and this relationalism thinking differs from the individualism of Western societies that emphasizes personal economic rationality [12,13,14]. In previous research [15,16] on trust theory in Taiwan, Wang, Chen, and Huang et al. [16] further used relationalism as a presupposition and proposed five major prototypes of trust strategies in Chinese society and local trust in Taiwan, demonstrating that the key factors in trust formation in Taiwan are significantly different from the trust theories in foreign countries.

These conceptual perspectives suggest that the key factors influencing trust in biotech brands should be culturally specific. Based on the concept of personal connections in Chinese relationalism and the trust strategies in Chinese society, this study provides further insight into trust in biotech brands through a long-term collaboration with Company X, a listed cord blood company in Taiwan. We participated in internal meetings held by the company, conducted in-depth interviews with product and supply chain actors and consumers, and finally conducted expert focus symposiums to identify the key factors that influence trust in biotech brands. Moreover, we constructed a structure model that affects consumer trust and purchase intentions and used survey research to collect data from target groups for empirical work. The objectives of this study are twofold. First, by introducing the concept of personal connections in Chinese relationalism and the trust strategies in Chinese society, this study aims to establish an initial framework for identifying and understanding the key factors that influence consumer trust and purchase intentions of biotech brands through long-term cooperation with Company X by means of participant observation, in-depth interviews, and focus group discussions. Second, this study will issue questionnaires to potential consumers of cord blood products in Taiwan through investigation and research methods and conduct statistical verification and analysis using structural equation models. Therefore, the purpose of this study was to develop an initial framework to explain the determinants of consumers’ trust in biotech brands and purchase intentions. And we performed statistical validation using structural equation modeling. The study hopes to propose marketing communication methods for health care products in different cultural contexts through the introduction of personal connections in Chinese relationalism and trust strategies.

## 2. Literature Review

### 2.1. Consumers’ Perceived Trust in Biotech Brands

Since the study of interpersonal interactions, trust has been frequently mentioned in many different fields and is considered an important concept, especially in risky situations such as economic transactions [7]. The definition of perceived trust varies across disciplines and perspectives, and trust is placed in different conceptual positions in diverse theoretical frameworks and analyzed at different levels, including macro, meso, and micro arguments [16]. However, in general, the scope of discussion on consumer perceived trust tends to be the context in which the transaction occurs alongside the characteristics of the trusted and the trustor.

A biotech brand needs to possess relevant attributes to be trusted. Kasperson et al. (1992) suggested that organizational assurance, competence, care, and predictability can be used to measure trust [17]. Peters et al. (1997) suggested measuring trust in three dimensions, namely general knowledge and expertise, openness and honesty, and care and attention [18]. Burnett et al. (2008) revealed in their study on organizational trust that trust in organizations is mostly correlated with professional competence such as experience, reliability, and competence of the organization itself, and individuals’ perceptions of whether the organization has the professional competence to fulfill its responsibilities will affect the level of trust in the organization [19]. The past studies indicate that to be a trusted organization, its professional competence, fairness, reliability, goodwill, and honesty impact trustworthiness.

Based on the past studies [20,21,22], this study defined the consumers’ trust in biotech brands as consumers will have more trust in the biotech brand when they feel professional and reliable about the biotech brand during shopping, that the brand treats consumers with goodwill, and that they will be treated honestly and fairly during the transaction. In previous studies on the characteristics of the trustor subject, it was indicated that trust involves the trustor’s own psychological state, and therefore, there may be varying responses and outcomes under different individual characteristics [23]. Trust is influenced by the trustor’s individual characteristics, and individuals and groups differ in trust formation mechanisms, resulting in different trust responses under uncertainties [24]. In addition, Gefen et al. suggested that the trust formation mechanism of consumers varies with their individual characteristics [7]. Therefore, to fully understand the trust relationship between consumers of biotech products and biotech brands in Taiwan, the trust formation mechanism of consumers must also be taken into consideration. The mechanism should be influenced in line with trust development in our culture, which differs from that of Western consumers; thus, the trust mechanism of Western consumers should not be fully copied onto our consumers.

### 2.2. The Formation Mechanism of Consumer Brand Trust in Taiwan

In previous literature on trust formation mechanisms, there are two main approaches to the discussion [7]. The first approach uses a structural framework that integrates specific beliefs (referring to a particular basis of trust) and general trust (referring to related concepts of trust, such as reliability and goodwill) in consumers’ perceived trust [25]. The other approach treats specific beliefs as antecedents of general beliefs and views trust as a process of integration of specific beliefs, that is, consumers use specific beliefs to form general beliefs with the intention of trust [26]. The latter approach to trust discussion can be a way to clearly depict consumer’s trust formation mechanism and usage intention in the context of the lack of trust antecedents in the biotech industry [7,26,27,28,29]. Therefore, the present study adopts the latter approach to the trust formation mechanism and uses specific beliefs as the antecedent of trust formation to explain the formation process of general trust intentions.

In terms of trust theory, the biggest difference between the Chinese trust formation mechanism and Western culture is that the key to trust in Chinese society lies in the relationship between the trustor and the trusted, whereas in Western society, it is the individual’s personal experience towards a specific activity. Luo et al. [12] have argued that Chinese “guanxi” differs from the relationship discussed in the West under social ties and have classified Chinese relationships into four categories: no ties, weak ties, acquaintance ties, and family ties, and this classification concurs with the “relationship” networks Bian et al. developed in 1997 for China and Singapore [30]. The Chinese trust relationship is one in which the trustor transforms under such different ties and identifies the “relationship” between the two parties as the basis for trust formation [12]. This also shows that in the Chinese development of trust, the concentric relationship of closeness, or relationalism [13], has been embedded in the structured foundation of trust.

According to studies conducted by Lo and Yeh from 2000 to 2007, Chinese trust in economic activities can be regarded as a concept of personal connections [12]. Such a concept of personal connections differs from the Western concept of relationship, which is an egocentric network, and therefore, such a concept of personal connections is different from the Western concept of trust under social capital, which is an egocentric social network that exists in the subjective consciousness of individuals. With this concept of personal connections, Chinese trust relationships in economic transactions can fall into three categories, such as near-family ties, acquaintance ties, and weak ties. The proximity of these three categories of trust relationships is distinguished by the proximity of the individual’s egocentric network and expands outward in a concentric manner. The weak ties of trust are mainly derived from social exchanges in transactions, acquaintance ties are primarily more than the weak ties with the exchange of human relationships, and near-family ties are mainly derived from blood ties and worship in minority Chinese cultures.

In a study, Wang, Chen, and Huang also proposed a similar concept using the relationalism methodology, suggesting that in the cultural context of Taiwan, the concept of trust can be divided into five major prototypes based on in-network and out-of-network [16]. The five major prototypes of local trust in Taiwan include the kinship trust and breeding bases emergent trust within the individual relationship network, as well as the professional foundation professional trust, institutional foundation institutional trust, and custom foundation customary trust outside the network, and suggest that the trust bases within and outside the network will coexist and determine the trust relationship between the two parties for specific targets [14]. Among these five major prototypes, the concept of kinship trust within the network is similar to that of family ties, while the emergent trust includes social exchanges and the exchange of human relationships arising from the interactive relationship, which implies weak ties and acquaintance ties under the concept of family ties. By adding professional trust, institutional trust, and customary trust outside of the relationship network as the basis of trust formation, we further explained the formation mechanism of local trust in Taiwan.

For the relationship between Taiwan’s consumers and biotech brands, since almost all economic behaviors are carried out in a fixed contract and institutional relationship, such a mode of relationship should not be similar to kinship trust and exchange of human relationships under near-family ties or acquaintance ties, and it is less likely to generate emotional considerations [7]. Even with the weak ties under social exchange, in the consumption context of Taiwan’s biotech products, only the part of economic exchanges often remains, which is included in the basic concept of the system in the five prototypes [1]. Therefore, the kinship trust and emergent trust of the five prototypes of local trust are less likely to occur in the trust of Taiwanese consumers in biotech brands. However, whether the trust prototypes within these relationship networks affect the formation of consumer trust in biotech brands in a transferable manner, and which relevant actors within the relationship networks influence Taiwanese consumers when purchasing biotech products, are questions that this study is eager to investigate. In addition, since biotech brands in Taiwan have been developed for only a decade, there is no possibility that they are passed on from history or preceded by norms for consumers. Consequently, trust relationships arising from customary trust should not exist in the consumer market context of the biotech industry in Taiwan [14,15]. Overall, this study suggests that through trust transfer, kinship trust and emergent trust within the consumer relationship network will likely influence the formation mechanism of Taiwanese consumer trust in biotech brands and when combined with two prototypes such as professional trust and institutional trust outside the consumer relationship network, they together form the specific antecedents of Taiwanese consumer trust in biotech brands and make effective predictions of general trust. These two trust construction mechanisms are further explained below.

#### 2.2.1. Trust Transfer Mechanism of Biotech Brands in Consumer Relationship Network

Wang and Liu conducted a trust analysis on Chinese people facing different roles, and the results showed that when facing different targets, Chinese people trust their family members the most [12]. Wang also cited data from a study conducted by the Association for the Advancement of Ethics, which showed that Taiwanese people trust their family members as high as 96.1% [14]. This evidence demonstrates that even in today’s Chinese society, the most important basis for developing trust relationships comes from blood, and sometimes family members are even a guarantee for the trustors themselves [12]. In the trust theory proposed by Wang, kinship trust generated by blood relations is a constant relationship under genetic continuity, and the trustor has a high sense of obligation or expectation to the trusted, even ignoring the consideration of risk. Such trust exists in the form of a life community in the trust construction of an individual [16]. On the basis of such kinship trust, if the parents, brothers, or other blood relatives of Taiwanese biotech product consumers can trust a certain biotech brand, feel assured about the purchase of biotech products from that biotech brand, and even recommend it, the process of trust transfer should make it possible to transfer the kinship trust to the biotech brand [25]. Therefore, this study concludes that through the transfer of trust to the biotech brand based on kinship trust, the trust development of biotech brands in Taiwan should be positively related to the trust of their blood relatives in the biotech brand.

In Chinese economic transactions, acquaintance ties are second only to blood ties for trustors and have some influence on them, such as acting as introducers to establish minimal trust relationships [12]. The influence of reference groups in social networks on individuals has also been discussed in previous theories of consumer behavior, such as the Theory of Rational Behavior and the Theory of Planned Behavior [31]. Taken together, these concepts suggest that trustors are influenced by their acquaintances and reference groups while establishing trust relationships with the trusted person and will make trust judgments under such influence. In the concept of the emergent trust in the local trust model, the emergent trust generated by emotional relationships is a lasting relationship under the sense of belonging and recognition, and the trustor has a high sense of obligation or expectation toward the trusted person, thus influencing the individual’s subjective judgment of risk and existing as a form of commitment and recognition in the individual’s trust construction [14,15]. Therefore, if an acquaintance or friend can trust a biotech brand, use it, or even recommend it, through the process of trust transfer, consumer trust in acquaintances and friends should be transferred to their trust in biotech brands. Consequently, this study also suggests that, through the transfer of a emergent trust, the development of consumer trust in biotech brands in Taiwan should be positively correlated with the trust of their acquaintances and friends in the biotech brands.

#### 2.2.2. Factors Influencing Trust in Biotech Brands outside the Consumer Relationship Network

In contrast to the influence of kinship trust and emergent trust on the formation mechanism of consumer trust in Taiwan, which is formed through the transfer of relationship networks, professional trust comes from the ability of the trusted person to resolve difficulties and crises faced by the trustor with professional competence [14]. Previous discussions on the characteristics of the trusted person reveal that the trust of the trustor in the organization is mostly related to the reliability and professional competence of the organization itself [17,18,19], indicating that professional trust should be an essential and fundamental characteristic of the trusted person. Professional trust is a type of authentication and analysis based on the organization’s expertise to predict the organization’s professional competence to respond to changes in the environment, and the trustor has less expectation of the trusted person and measures risk by a predictable standard that survives through dialectic and analysis [14]. If a biotech brand has a high level of professional competence, consumers will have more confidence in its ability to solve problems in the future. In other words, if a biotech brand has a high level of expertise, consumers will have more confidence in its ability to solve problems in the future, and this will enhance consumer trust in the biotech brand through the prediction process and competence process of the trust formation mechanism. Therefore, this study concluded that if the professional capabilities of the biotech brand are more recognized by consumers, consumer trust in it will increase positively.

Institutional trust is defined as a written or unwritten system that enables trustors and the trusted person to interact with each other after rationally calculating their own interests, in which the system can serve as a norm and a rule of the game to reduce uncertainty and risk in the risky market [14]. Institutional trust generated by interest relations is a relationship that changes with the context of time and space under the continuation of legalization and morality. The trust of the trustor to the trusted person comes from an objective risk consideration and exists in the form of a contract or regulation in the individual’s trust development. In particular, in the context of the time risk of many products in the biotech industry, if a biotech brand can provide consumers with clear and strong protection through corresponding systems and regulations, the risk of consumers’ transactions with the biotech brand can be reduced. Such a concept is roughly the same as the concept of institutional trust proposed by Gefen et al. [7]. By trusting the relevant regulations of a biotech brand, consumers can reduce their transaction costs with the company and reduce their risk perception in an uncertain environment. A consumer’s trust in a biotech brand will increase if he/she perceives that the brand has a complete system.

Combining the above two factors, this study suggests that consumer trust in biotech brands in Taiwan is significantly and positively influenced by their family and acquaintances within their relationship networks, as well as by the institutions and level of expertise outside their relationship networks. If consumers perceive that their family and acquaintances have a higher level of trust in the biotech brand, such trust will influence their trust in the biotech brand through transfer and reference mechanisms that generate a base of trust within the relationship network. If consumers perceive that the biotech brand has better institutional protection and higher professional competence, the institution and professional competence will influence their trust in the biotech brand through direct influence mechanisms that generate trust outside the relationship network. In addition, since previous studies have not defined which acquaintances influence consumers of biotech products, this research team will work closely with Company X to identify the role of acquaintances in the consumer relationship network of the biotechnology industry realistically, allowing the results to be directly applied in practice. Likewise, we have also considered other dimensions of the biotech industry in Taiwan, so that the relevant insights can be fully applied to future marketing priorities.

### 2.3. The Impact of Trust in Biotech Brands on Consumer Purchase Intention

In previous studies, the influence of trust on behavioral intentions has been investigated across disciplines. Whether from the perspectives of motivation [32], goodwill [33], expectation [34], rational calculation [35], and fairness [36], or from the concepts of reliability, goodwill, fairness, and integrity, it has been suggested that individual trust can influence individual behavioral intentions. Rousseau et al. [16] have compiled past literature on trust across disciplines and have argued that trust is the act of voluntarily engaging in a hurtful situation because of a positive expectation of another’s intentions or behaviors. Siegrist and Gutscher made a similar argument, arguing that trust is the belief in the reliability of another person in a risky situation when making behavioral decisions under uncertainty [3]. In other words, trust can be described as a voluntary dependence in the process of an individual’s transaction with a counterparty. In the process of trading between an individual and a counterparty, trust can be described as a voluntary dependence on the willingness to put oneself in a vulnerable and uncertain situation to make a purchase [37].

These definitions reflect that trust often occurs when the trustor is in an uncertain or even risky situation but still holds a positive expectation of the trusted person and is willing to bear the possibility of being harmed. With most biotech products today emphasizing future applications, consumers are in a time-risk situation when making a purchase, and consumer trust in biotech brands has a profound impact on the smoothness of the transaction between them. In previous studies on risky situations, it is generally believed that if consumers can feel trust in the risky situation and thus reduce the perceived risk of shopping, it will increase consumers’ purchase intention [7,8,38]. Related studies have also demonstrated that consumers are willing to pay higher prices for transactions with manufacturers when they are perceived to be trustworthy because their performance agrees with the ethical standards and behaviors of society, whereas manufacturers failing to increase consumer trust has a negative impact on consumers’ intention to use the product [2]. Thus, in the consumer behavior model proposed by Gefen et al. [7], trust plays a pivotal role in the influence mechanism of consumer purchase intention in risky situations, which is a key factor that directly affects purchase intentions as well as a mediator of other factors in purchase intentions [7]. Therefore, this study also suggests that consumer trust in a biotech brand positively affects their willingness to purchase the brand, that is, the higher the consumer’s perceived trust in a particular biotech brand, the higher the consumer’s purchase intention for that brand’s biotech products.

### 2.4. Structure Model of the Study

**Figure 1:**
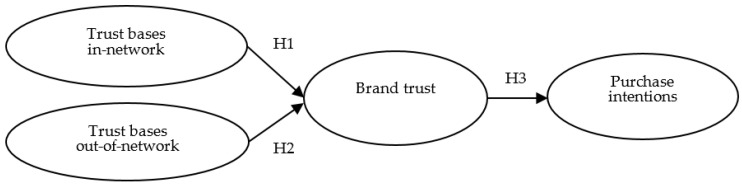
Structure model of the study.

In this study, three key points are summarized in the literature to better understand consumers’ purchasing behaviors toward products related to the biotech industry. Firstly, with respect to the mechanism of developing brand trust in Taiwan, we argue that consumers in the biotech industry develop brand trust by transferring and referring to family members and acquaintances within the relationship network and incorporate institutional and professional considerations outside the relationship network to jointly construct consumers’ imagination of trust in biotech brands. Secondly, previous studies have only focused on the concept of trust and not on the special characteristics of the biotech industry, and there is no mention of the concept of acquaintances of consumers in the biotech industry in Taiwan. Thus, we will conduct in-depth interviews and focus group discussions to understand the biotech industry in Taiwan, construct a prototype of the relationship network of consumers in the biotech industry in Taiwan, and then conduct empirical evidence. Finally, based on relevant surveys and previous literature, this study concludes that trust is a major factor affecting consumer purchase intention in the biotech industry (Figure 1). Therefore, after compiling the above arguments, this study proposes the following conceptual or structure model based on prior studies [15,16] in the hope to make concrete contributions to both theory and practice through a qualitative and then empirical research approach. Three hypotheses were proposed based on prior studies as follows. Two structures, including a measurement model (i.e., Confirmatory Factor Analysis; CFA) and a structural mode, in which multiple equations could be estimated simultaneously with four latent constructs.

**Hypothesis** **1** **(H1).** *Consumers’ trust bases in the social network positively influence their trust in the biotech industry*.

**Hypothesis** **2** **(H2).** *Consumers’ trust bases out of the social network positively influence their trust in the biotech industry*.

**Hypothesis** **3** **(H3).** *Consumers’ brand trust in the biotech industry positively affect their purchase intentions*.

## 3. Materials and Methods

### 3.1. Questionnaire Design

The empirical part of this study was conducted through a structured questionnaire using a survey research method. To bridge the gap between theory and practice and align the findings with the current industry status as much as possible, the research team conducted a series of intensive meetings with Company X prior to the formal design of the questionnaire. This was done to identify the different roles that influence consumers in the biotech industry within the social network and advance the understanding of the institutional and professional dimensions outside the social network.

According to the trust theory in Taiwan proposed by Wang et al. [15], we used the relationalism methodology and divided the concept of trust to in-network and out-of-network bases. In terms of the foundation of trust within the consumer social network, through the results of the meetings and interviews with Company X and the consumers, this study identified four major roles of consumers within the social network when purchasing biotech products: family members, acquaintances (including other relatives and friends), health care workers, and netizens. A pilot study was used to first collect above stakeholders’ opinions. To enhance the validity of the questionnaire, the research team further conducted a focus group discussion between consumers and government-industry-university to confirm the significance of these roles in creating consumer trust in the purchase of biotech products. Four main semi-structured interview questions were addressed among focus group, including (1) What are consumers’ primary sources of information about the cord blood products? (2) How does a cord blood company establish an interactive relationship with consumers? (3) What factors will influence consumers’ decision making before purchasing cord blood products? (4) What are the consumers’ trust and overall evaluations of one cord blood products? At the same time, we adjusted the text of the questionnaire related items in response to the discussion outline to better suit the consumer’s language. In the end, 12 questions (three questions for consumers of family members, acquaintances, health care workers, and netizens) were designed to measure the basis of trust generated by consumers through the relevant key players in their social networks.

As for the trust base outside the consumer’s social network, this study referred to the intent of relevant domestic and overseas trust theories and considered the current situation in Taiwan. Based on the questionnaire designed in empirical studies in Taiwan [8], the questionnaire was modified according to the concepts of the trust theory proposed by Wang [14] to lay down questions related to the foundation of trust outside the network, such as institutional trust and professional trust. Similarly, the research team proposed relevant questions for discussion with consumers and government-industry-university experts in a focus group session, and finally developed questions related to institutional trust and professional trust (three questions each).

This study referred to the main design implications of dimensions in Davis’s technology acceptance model [39] and incorporated the interaction concepts mentioned in the integration model proposed by Gefen [7]. After considering the current situation of the biotech industry in Taiwan, the trust and intention scales adopted by Wu et al. were used as the framework [8] to construct the initial questions of brand trust (four questions) and purchase intention (three questions). Likewise, the research team also developed questions through meetings and focus groups, taking into account the opinions of consumers and government-industry-university experts to enhance the validity of the content.

After establishing the content validity of the questionnaire through meetings, in-depth interviews, and focus groups, pre-testing was also conducted. Among the 54 pre-test samples, the Cronbach’s α values of each dimension, including family members, acquaintances, health care workers, and netizens within the relationship network, institutional and professional bases outside the relationship network, and brand trust and purchase intention, exceeded 0.7, which met the standard of internal consistency, indicating that the questionnaire in this study had a certain level of reliability. As for the test of convergent validity, this study conducted a pretest validity analysis employing principal component analysis and revealed that only a single component was extracted from each dimension when the eigenvalue was greater than 1, that is, the explanatory variance of each dimension was represented by a single component at a certain level, indicating that the design of this questionnaire has a certain degree of convergent validity.

PLS was also used to confirm the model reliability and validity in advance, and it was found that both the negative factor loadings and the significant pathways met the conceptual expectations, which further confirmed the model reliability and calibration validity of this questionnaire. Finally, a total of 25 questions were included in the questionnaire: family members (three questions), acquaintances (three questions), health care workers (three questions), and netizens (three questions) within the consumer’s social network, institutional base (three questions) and professional base (three questions) outside the social network, and brand trust (four questions) and purchase intention (three questions). The questions were scored on a seven-point Likert scale in the order of strongly agree (seven points), agree (six points), somewhat agree (five points), no opinion (four points), somewhat disagree (three points), disagree (two points), and strongly disagree (one point) to represent consumers’ level of agreement with the question. To avoid the effect of common method variance or common method bias, the formal questionnaire was designed to present a random number of questions and was tested through Harman’s single factor testing. The findings indicated that the total variance through the principal component explained 41.65%, indicating that there was no common variance in the results of the questionnaire.

### 3.2. Data Collection

To understand the purchasing behavior of potential consumers in the biotech industry, especially their trust-building mechanism, this study utilized a questionnaire distributed at Mom’s Class (two in Taipei, one in Taichung, one in Kaohsiung, and one in Hualien) and Baby & Mommy Expo (one in Taipei, one in Taichung, and one in Kaohsiung) at hospitals in Taipei, Taichung, Kaohsiung, and Hualien during the three months. A total of 300 questionnaires were distributed, and 181 questionnaires were collected from women who had heard of Company X. In addition, this study also used online questionnaires for distribution in the discussion, published announcements in the discussion boards of major biotech companies, pregnant mother communities, and various communication software, and used lotteries and rewards to enhance consumers’ willingness to fill out the questionnaires. Therefore, 261 questionnaires were collected in this study, and after excluding five invalid samples, the final valid sample of this study was 239.

For the basic data of the collected samples, we compared the sample data with the customer database of Company X and confirmed that the valid samples of this study were close to the existing customer data in terms of age structure, education level, and household income. Further, to confirm the stability of the structure model, the PLS model was calculated for both the in-person and online samples, and the model results and predictive power of both subsamples were found to be similar to the overall sample, demonstrating a certain level of the stability of the structure model.

## 4. Data Analysis

In this study, SPSS (version 23.0, IBM, Armonk, NY, USA) and AMOS (version 22.0, IBM, Armonk, NY, USA) were used as statistical software to analyze the sample data. Judging from the results of the internal consistency analysis, the Cronbach’s α values of each dimension of the study and the questionnaire as a whole exceeded 0.7, falling between 0.859 and 0.958, which met the criteria of internal consistency (Table 1). This indicated that the data in this study have good reliability and are suitable for the operation of arithmetic averaging within the dimensions.

A structural equation model was used as the validation method for the hypothesis. After confirming the internal consistency of the family base, acquaintance base, health care worker base, and network base within the relationship network, as well as the institutional base and professional base outside the relationship network, the dimensions were aggregated through arithmetic averaging to avoid statistical bias caused by too many estimation parameters and to improve the validity of the model.

### 4.1. Analysis Results of the Measurement Pattern

In the light of the results of the structural equation model, the questionnaire received good support in all reliability and validity tests. The factor loadings of all questions were in the range of 0.78–0.93 (Table 2), which satisfied the statistical standard of 0.5–0.95 recommended in the literature of structural equation modeling [40,41], indicating that the measurement pattern of the model has good internal validity and model reliability.

The measurement pattern of each dimension was further examined, and the results of the confirmatory factor analysis (CFA) were used to calculate the composite reliability (CR) and the average variance extracted (AVE) (Table 3). It has been suggested that CR values for each dimension should be greater than 0.7, and AVE values should be greater than 0.5. The CR values for each dimension in this study ranged from 0.870 to 0.919 and AVE values from 0.690 to 0.865, both of which agreed with previous scholarly recommendations, indicating that the scale performed well in terms of convergent validity. As for discriminant validity, the square root of the AVE and the correlation coefficient between the dimensions were compared (Table 4), and the correlation coefficients between the dimension were found to be less than 0.85 and smaller than the square root of the AVE, which accorded with the previous literature on discriminant validity [42], indicating that the scale has good discriminant validity.

### 4.2. Analysis Results of the Overall Model

The Goodness-of-Fit index (GFI) of the overall model adopted was recommended by Gefen et al. [7], Tan [43], and Kline [44], and the GFI, AGFI (Adjusted Goodness-of-Fit Index), RMR (Root Mean Square Residual), chi-square/degree of freedom (χ^2^/df), and NNFI (Non-Normed Fit Index) were used as comparison indicators. For the GFI and AGFI, Kline suggests that these two indices between 0.8 and 0.89 indicate reasonable goodness-of-fit, Doll et al. believe that these two indices above 0.90 indicate significant goodness-of-fit, and others deem a GFI greater than 0.9 and an AGFI greater than 0.8 implies that the SEM is acceptable [45]. In terms of RMR value, Doll et al. believe that a value lower than 0.05 means that the model had a reasonable goodness-of-fit, but some think that an RMR value lower than 0.08 exhibits that the model is acceptable [44]. The chi-square/degree of freedom is considered to be less than 345, and an NNFI value greater than 0.90 is an indicator of goodness-of-fit for SEM, with a larger value indicating a better goodness-of-fit [44,45].

The SEM results showed that the overall goodness-of-fit of the model was very appropriate. GFI reached 0.96, AGFI was 0.93, and NNFI was 0.99, all of which were higher than the recommended goodness-of-fit indices; conversely, the RMR was 0.022, which was lower than the threshold of 0.05, and the chi-square/degree of freedom was only 1.34, which was lower than the recommended goodness-of-fit value. Other indices and the related results are shown in Table 5, which demonstrates that the overall goodness-of-fit of the model meets the criteria of the relevant indices [37], showing quite a good overall goodness-of-fit of the model.

**Figure 2:**
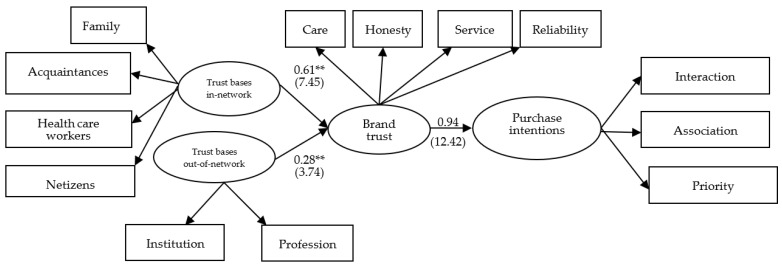
Structural equation model results of this study. Note: Standardized coefficients are outside the parentheses and *t*-values are inside the parentheses (Note: ** *p* < 0.01).

In the path analysis, the standardized coefficient of the influence of relevant actors in the social network on consumer trust of the biotech industry was 0.61 (*t* = 7.88, *p* < 0.001 ***), indicating that the higher the trust base in the social network, the higher the level of consumer trust in the biotech brand. In other words, family members, acquaintances, health care workers, and netizens in the social network of consumers in the biotech industry significantly and positively influence consumer trust in biotech brands. As for the influence of institutional and professional bases outside the social network, the standardized coefficient of the influence of trust bases outside the social network on consumer trust in the biotech industry was 0.28 (*t* = 3.74, *p* < 0.01 **), which indicates that the higher the trust bases outside the social network, the higher the consumer trust in the biotech brand. Thus, the institutional and professional bases known to consumers in the biotech industry will significantly and positively influence the level of consumer trust in biotech brands. Moreover, the standardized coefficient of 0.94 (*t* = 12.41, *p* < 0.001 **) for the effect of consumers’ brand trust in biotech brands on their purchase intention indicates that the higher the brand trust in biotech brands, the higher the consumer purchase intention for the biotech brands. As a result, the level of consumer trust in the biotech industry will significantly and positively affect their purchase intention of biotech brands. Last, the results of collinearity diagnostics show that all coefficients of Variance Inflation Factor (VIF) ranged from 1.35 to 1.68 are less than five, indicating acceptable level of the multicollinearity between three constructs [46]. A summary of the testing results of the three hypotheses is presented in Table 6 suggested by prior studies [47,48]. Figure 2 shows graphically the resulting path coefficients of the overall structural model.

## 5. Conclusions and Discussion

### 5.1. Conclusions

Today, with the rapid take-off of the biotech industry, brand trust has become a must for biotech brands in practice. However, the dispute over trust in biotech brands has lacked empirical support in the past, and the previous understanding of trust in Taiwan’s academic research was not profound. Therefore, this study attempted to provide an academic direction for the development of biotech brands in Taiwan through a two-pronged approach of qualitative exploration and quantitative empirical evidence while addressing study limitations. Specifically, the findings of this study are the following three.

First, this study introduced the Chinese concept of personal connections [12] and local trust theory [14] into the construction mechanism of trust in biotech brands through a review of previous literature. We sorted out the antecedents of Taiwanese consumer trust in biotech brands within and outside of their relationship networks, examined each of the five prototypes of local trust theory, and finally focused on the transfer of trust from family and acquaintances within the relationship networks of consumers in the biotech industry, as well as on the institutional and professional influences outside the relationship networks, thereby constructing the model of this study. Consequently, by introducing the concept of personal connections and local trust theories, this study overcame the shortcomings of the previous discussion of biotech consumers in Taiwan and proposed to understand the trust construction mechanism of biotech consumers in Taiwan by examining both inside and outside the relationship network. The study also suggested that consumer trust in a particular biotech brand can significantly and positively influence their intention to purchase that biotech brand.

Next, we worked closely with Company X to conduct a dozen internal meetings and external interviews. That helped our research team to clarify the structure of the biotech industry in Taiwan and enhanced our understanding of the institutional and professional bases outside the relationship network of biotech consumers. Furthermore, we used participant observation and in-depth interviews as qualitative research methods to extract the key players in the relationship network that affects biotech consumers in Taiwan. The research team then used focus groups to combine the strengths of a dozen consumers and government-industry-university experts to discuss in depth the results of our qualitative research in the first half of the year. The important roles in the relationship network were identified in four categories: family members, acquaintances (including other relatives and friends), health care workers, and netizens, and the preliminary draft of the questionnaire was thoroughly examined in all aspects to enhance the content validity of the questionnaire. In the end, this study reconfirmed the related hypotheses obtained through the pre-test of 54 questionnaires and the statistical analysis, thereby establishing the reliability and validity of the relevant dimensions of this questionnaire.

Finally, the study collected samples in two different ways. First, we collected questionnaires from women who had heard of Company X at Mom’s Class and Baby & Mommy Expo in Taipei, Taichung, Kaohsiung, and Hualien. In addition, we also distributed online questionnaires, and finally, we obtained 239 valid samples. We adopted AMOS to analyze the structural equation model, and the CFA results of the measurement pattern showed that all dimensions of the questionnaire met the criteria in terms of factor loadings, CR values, AVE values, and discriminant validity, which again verified the reliability and validity of the questionnaire. In respect of the goodness-of-fit of the overall model, this study also met the criteria of the previous structural equation model [45], indicating that the model can be empirically validated. This implies that our proposal that the transfer of trust from family and acquaintances within the relationship network of biotech consumers and the direct influence of profession and institution outside the relationship network jointly construct the trust of biotech brands in Taiwan, which in turn affects consumer purchase intention, is fully supported. The path analysis results further showed that the behavioral intentions of consumers in the biotech industry in Taiwan are basically related to their brand trust, and to construct such a brand trust, the transfer of trust within the consumer relationship network is more crucial than the institutional and professional bases outside the relationship network.

### 5.2. Discussion

The model of this study is supported by empirical data, which makes it possible to introduce the trust theory of relationalism into the understanding of consumer trust in Taiwan, and the dimensions of the consumer relationship network extracted from the in-depth interviews and focus group discussions by the research team are also relevant to the current status of the biotech industry in Taiwan. The findings of this study are further discussed from theoretical and managerial implications.

For theoretical contributions, the empirical findings fill in the gaps in the previous understanding of trust in biotech brands in Taiwan and provide a high correlation between trust in biotech brands and purchase intention and the construction mechanism of trust in biotech brands. Specifically, previous studies on consumer trust have often only replicated Western trust theories onto Taiwanese consumers, and the current development of non-Western trust theories has only broken through in qualitative research but has not yet conducted empirical studies with empirical evidence [12,13,14,15]. This study discussed trust theory in a relationalism context and considered the relationship network of biotech consumers as the core concept. It is advocated that a two-pronged approach should be adopted in the construction of brand trust in the biotechnology industry in Taiwan, with the transfer of family and acquaintances within the relationship network and the influence of institutions and professions outside the relationship network to jointly construct consumers’ trust in biotech brands. This study has successfully changed the perspective on consumer trust and has also developed a credible and reliable study through actual industry roots and pre-testing. By clarifying the connotations inside and outside the relational network through qualitative interviews for more than six months, we not only succeeded in changing the perspective of consumer trust but also formed a reliable questionnaire through actual industry roots and pre-tests and then supplemented the empirical evidence of relational trust theory through empirical research. Therefore, this study not only incorporates the concept of relationalism into the development of trust theory of consumer behavior in Taiwan but also provides a clearer picture of the way consumer trust is constructed in specific industries through systematic observation inside and outside the relational network, thus adding to the empirical evidence of relationalist trust and providing a reference for future consumer behavior research.

In terms of management implications, this study uses consumer relationship networks as the core of trust in biotech brands, and three insights can be drawn from the empirical findings. First, if a biotech company is trying to build trust in its brand, it should adjust its brand positioning so that consumers perceive it as not only focusing on their personal interests but also caring about other key factors inside and outside the consumer relationship network. In the past, this may have been an accidental attempt, but the results of this study show that this is an empirical based approach. Second, important players within the consumer relationship network can influence the trust construction of biotech brands through trust transfer mechanisms, and the effect is greater than that of factors outside the relationship network, which is a significant difference from the current institutional and professional emphasis of biotech brands in practice and requires special attention. Therefore, we suggest that when biotech brands try to build consumer trust, they should prioritize the care and demands of consumers’ family members, acquaintances (including other relatives and friends), health care workers, and netizens. For example, strengthening B2B marketing efforts for medical professionals and even hospitals or creating a positive image in specific mom communities may have a key impact on consumer trust in the brand. Third, institutions and professions outside of the consumer relationship network, while less influential than key players within the relationship network, still have a significant impact on consumer trust. In practical terms, therefore, how to make consumers perceive a more complete system and more reliable profession will make consumers feel more secure and trustful. In addition to the more common listing and scale assurance and professional certification, strategic alliances in the biotech industry can increase consumers’ imagination of scale and professional association at the same time and may be a good way to enhance brand trust.

## Figures and Tables

**Table 1:** Reliability analysis of the questionnaire.

	Cronbach’s α		Cronbach’s α
Family base	0.895	Brand trust	0.921
Acquaintance base	0.938	Purchase intention	0.871
Healthcare worker base	0.891	Overall model	0.958
Netizen base	0.923		
Institutional base	0.859		
Professional base	0.893		

**Table 2:** Factor loadings for each dimension.

	Factor Loading		Factor Loading
Trust base in-network		brand trust	
Family base	0.78	Customer care	0.82
Acquaintance base	0.87	Honest dealing	0.82
Healthcare worker base	0.82	Good service	0.83
Netizen base	0.91	Trustworthy	0.89
Trust base out-of- network		Purchase intention	
Institutional base	0.92	Willingness to interact	0.75
Professional base	0.93	Demand association	0.87
		Priority purchase	0.81

**Table 3:** Composite reliability and average variance extracted.

Dimension	CR	AVE
In the relationship network	0.910	0.716
Out of the relationship network	0.922	0.856
brand trust	0.906	0.706
Purchase intention	0.852	0.659

**Table 4:** Discriminant validity test.

Dimension	In the Network	Out of the Network	Overall Trust	Purchase Intention
In the relationship network	0.846			
Out of the relationship network	0.659	0.925		
brand trust	0.716	0.665	0.840	
Purchase intention	0.686	0.614	0.815	0.812

Note: Diagonal is the square root of the AVE value, whereas non-diagonal is the correlation coefficient between the dimensions.

**Table 5:** Overall goodness-of-fit indices for this study.

Index	Standard	Result
GFI	>0.9	0.96
AGFI	>0.8	0.93
NNFI	>0.9	0.99
RMR	<0.05	0.022
χ^2^/df	<3	1.34
NFI	>0.9	0.98
CFI	>0.9	0.99
IFI	>0.9	0.99
RFI	>0.9	0.99
PGFI	>0.5	0.59
PNFI	>0.5	0.71
SRMR	<0.05	0.023
RMSEA	<0.05	0.036

**Table 6:** Summary of the Tested Hypotheses 1–3.

Research Hypothesis	Hypothesized Path	Expected Sign	Path Coefficient	*t*-Value	VIF	Results
H1	Trust bases in the social network → Consumer trust in the biotech industry	+	0.61	7.88 **	1.77	Supported
H2	Trust bases outside the social network → Consumer trust in the biotech industry	+	0.28	3.74 **	1.76	Supported
H3	Consumer trust in the biotech industry → Purchase intentions	+	0.94	12.41 ***	2.37	Supported

** *p* < 0.01, *** *p* < 0.001.

## Data Availability

The data presented in this study are available on request from the corresponding author. The data are not publicly available due to containing information that could compromise the privacy of research participants.
